# Biochemical composition and antioxidant capacity of extracts from *Podophyllum hexandrum* rhizome

**DOI:** 10.1186/1472-6882-12-263

**Published:** 2012-12-22

**Authors:** Mengfei Li, Lanlan Zhou, Delong Yang, Tiantian Li, Wei Li

**Affiliations:** 1 Gansu Provincial Key Lab of Aridland Crop Science / College of Life Science and Technology, Gansu Agricultural University, Lanzhou, 730070, Gansu, P.R. China

**Keywords:** *Podophyllum hexandrum*, Biochemical composition, Antioxidant capacity, GC-MS

## Abstract

**Background:**

*Podophyllum hexandrum* Royle (*P*. *hexandrum*) is a perennial herb and widely used in clinic. The present study was designed to separate and identify the biochemical composition and antioxidant capacity of extracts from *P*. *hexandrum* rhizome.

**Methods:**

The ethyl acetate and ethanol extracts from *P*. *hexandrum* rhizome were analyzed by GC-MS (gas chromatography–mass spectrometry), and the antioxidant capacity of the extracts and the components was tested by using the DPPH (2, 2-diphenylpicrylhydrazyl) and FRAP (Ferric reducing/antioxidant power) assays.

**Results:**

The rhizome extracts had greater antioxidant capacity than the petiole extracts in DPPH and FRAP assays. About 16 kinds of main reactive oxygen components were identified in the extracts. Components of PADE (Phthalic acid, diisobutyl ester), BADE (1,2-Benzenedicarboxylic acid, diisooctyl ester), Polyneuridine, PODD (Podophyllotoxin, deoxy), β-Sitosterol and POD (Podophyllotoxin) showed the antioxidant capacity in some degree. PODD, POD, and Polyneuridine showed stronger antioxidant capacity with the IC_50_ and FRAP values of 9.61 ± 0.81 and 2923.98 ± 21.89 μM, 9.98 ± 0.24 and 2847.27 ± 14.82 μM, and 13.37 ± 0.35 and 2404.32 ± 36.88 μM, respectively, than the positive control ASA (Ascorbic acid) with the values of 60.78 ± 1.22 and 1267.5 ± 30.24 μM (*P* < 0.01).

**Conclusions:**

PODD, POD, and Polyneuridine are very critical for the antioxidant capacity in the extract of *P*. *hexandrum* rhizome. These results provide useful biochemical basis and information for the potential use of this plant.

## Background

*Podophyllum hexandrum* Royle (*P*. *hexandrum*), commonly named Himalayan Mayapple, is a perennial herb that grows in the Himalayan region and the southwest of China [1,2]. Since 1940 *P*. *hexandrum* resin has been used topically for various skin lesions such as warts and condylomas [3]. POD is a natural product mainly existing in *P*.*hexandrum* rhizome. It has been used in the treatment of genital infection to sterilize noncervical human papilloma virus [4]. Penile warts can be safely treated with 0.5–2.0% podophyllin self applied [5]. Goel *et al*. [6] reported a significant antitumour effect at subtoxic, well-tolerated, sequential doses of aqueous extract of *P*. *hexandrum*. POD is also used as starting compound for the chemical synthesis of etoposide (VP16-213) and teniposide (VM-26), and its congeners and derivatives have pronounced biological activity as anticancer, antineoplastic and anti-HIV drugs [7-9]. Researchers have recently paid attention to the antioxidant activity of POD and its derivatives, such as GP7OH, GP7H, GP7 and VP16 [10]. So far, there is no specific study on the biochemical composition and the antioxidant capacity of *P*.*hexandrum* rhizome extracts. In our previous study, GC-MS showed that the ethyl ether extract of *P*. *hexandrum* seed contained some bioactive components, such as Hexanedioic acid, Oleic acid, and Octadecanoic acid [11].

In the present study, we tested the antioxidant capacity of ethyl acetate and ethanol extracts in the rhizome and petiole of *P*.*hexandrum* by the DPPH and FRAP assays, separated and identified the biochemical compositions of the rhizome extracts by GC-MS, and then evaluated antioxidant capacity of some identified compositions with the parameters of IC_50_ and FRAP value.

## Methods

### Plant materials

*P*. *hexandrum* was collected from a forest (2,100 m) in HuiChuan, WeiYuan of Gansu Province, China, after the plant fruit ripened in September 2011. The plant was dried in shade under room temperature. The species was identified by Professor Yanling Qi (Gansu Provincial Academy of Agricultural Sciences, Lanzhou, Gansu, P.R.China). A voucher specimen (No.0209069) was deposited in the herbarium of College of Agronomy, Gansu Agricultural University, Lanzhou, Gansu, P.R. China.

### Reagents and instrumentation

DPPH, 2, 4, 6-tris (2-pyridyl)-s-triazine (TPTZ), PADE, BADE, Polyneuridine, PODD, β-Sitosterol, and POD were purchased from Sigma Chemical Company (St. Louis, MO, USA). Ethyl acetate, ethanol, methanol, ASA, FeCl_3_ · 6H_2_O, and HCl were purchased from Guangfu Chemical Research Institute (Tianjin, P.R.China). UV-1810 (Beijing Persee General Instrument Co., Ltd, P.R.China) and Trace DSQ GC-MS (American Finnigan Company, USA) were used.

### Preparation of extracts

Rhizome and petiole were washed, dried and grinded to powder. Then the powder was weighted (20.00 g) and soaked in ethyl acetate and ethanol (500 mg/mL) for 10 months at room temperature in dark, and was then filtered, evaporated and condensed to dryness under nitrogen to obtain extracts.

### Antioxidant assays

Although there are numerous methods for determining the antioxidant capacity of soluble natural extracts and insoluble food components [12], no perfect system is available to help us know the “true” antioxidant capacity of a complex medium [13,14]. The DPPH and FRAP assays, despite their disadvantages, are still used by many researchers for rapid evaluation of antioxidant [15].

### DPPH assay

The free radical scavenging activity (FRSA) of DPPH was measured according to Ramadan *et al*. [16] and Nencini *et al*. [17]. which is one of the few stable and commercially available organic nitrogen radical assays [18,19]. Foti *et al*. [20] suggested it is an electron transfer reaction. The initial electron transfer occurs very quickly, while the subsequent hydrogen transfer occurs more slowly and depends on the hydrogen-bond accepting solvent [18,19]. This reaction has been measured by the decoloration assay where DPPH has an absorption band at 515 nm which disappears upon reduction by an antiradical compound [18,21]. The specific steps are as follows.

The extracts were diluted with 15% aqueous ethanol with concentration of 10 mg/mL, and then 50 μL of the diluted extracts was added with 950 μL of 10^−4^ M DPPH methanol solution. Then the mixture was shaken and kept in dark for 30 min at room temperature. The decreased absorbance of DPPH solution was evaluated at 515 nm by a spectrophotometer UV-1018, and 500 μM 15% aqueous ethanol ASA was tested as a positive control. The test was carried out in triplicate, and the capability to scavenge the DPPH radicals was calculated as:

(1) $$\begin{array}{l} \text{Scavenging}\;\text{effect}\left(I\%,\text{Percentage}\;\text{of}\;\text{inhibition}\right)\\ \;=[(A_{0}-A)/A_{0}]\times 100 \end{array}$$

where *A*_0_ and *A* were the absorbance of DPPH without and with sample, respectively.

### FRAP assay

The FRAP test was first introduced by Benzie *et al*. [22] for measuring the total antioxidant activity, which was initially developed to assay plasma antioxidant capacity but can also be used on other fluids. In the FRAP test, reductants (antioxidants) in the sample reduce ferric-tripyridyltriazine complex (Fe^3+^- TPTZ), in stoichiometric excess, to a blue ferrous form (Fe^2+^), with an increase in absorbance at 593 nm [17]. The specific steps were described by Tsao *et al*. [23] as follows.

The working FRAP reagent was prepared *ex* tempore by mixing 10 volumes of 300 mmol/L acetate buffer, pH 3.6, with 10 mmol/L TPTZ in 40 mmol/L HCl, and 20 mmol/L FeCl_3_ · 6H_2_O at 10:1:1 (v/v/v).

The 300μL FRAP reagent and the 10μL standard samples (FeSO_4_ · 7H_2_O, 500 μM) or test samples (10 mg/mL 15% aqueous ethanol) were added and mixed well. The reaction temperature was 37°C and the absorbance readings were taken at 593 nm immediately and 4 min later using a spectrophotometer UV-1018. And 500 μM 15% aqueous ethanol ASA was tested as a positive control. All tests were carried out in triplicate. The FRAP value of the test samples was calculated on the basis of 500 μM Fe^2+^ (FeSO_4_ · 7H_2_O) as follows:

(2) $$\text{FRAP}\;\text{value}\;\left(\mathrm{\mu M}\right)=(ÄA_{593\text{nm}}\text{test}\;\text{sample}/ÄA_{593\text{nm}}\text{standard}\;\text{sample})\times 500(\mathrm{\mu M})$$

where *ÄA*_*593nm*_ was the absorbance of the sample minus the absorbance of the blank at the 4th minute.

### GC-MS analysis

The samples were the rhizome extracts of ethyl acetate and ethanol (500 mg/mL). Trace DSQ GC-MS from American Finnigan Company was used. The GC conditions were: 1 N NOWAX quartz capillary column: 30 m × 0.32 cm × 0.25 mm; column temperature: 50–190°C; procedure temperature: 5°C/min; carrier gas: He; vaporizer temperature: 280°C; the MS conditions were: Ion source: EI; temperature: 200°C; ionizing voltage: 70 eV; electric current of collection: 300 μA; electric current of emission: 1 mA; resolution: 600; mass: 10–600.

### Evaluation of antioxidant assays

Reagents PADE, BADE, Polyneuridine, PODD, β-Sitosterol, POD, and including the positive control ASA were dissolved in 15% aqueous ethanol and tested at the same concentration of 500 μM. Antioxidant capacity was expressed IC_50_ (μM) and FRAP (μM) and the test methods were mentioned above (as the DPPH and FRAP assays).

### Statistical analysis

The results were presented as mean ± SD of triplicate determinations. Statistical analysis was performed by SPSS 11.5. One-way analysis of variance (ANOVA) was utilized to evaluate differences.

## Results

### Antioxidant capacity of rhizome and petiole extracts

**Figure 1:**
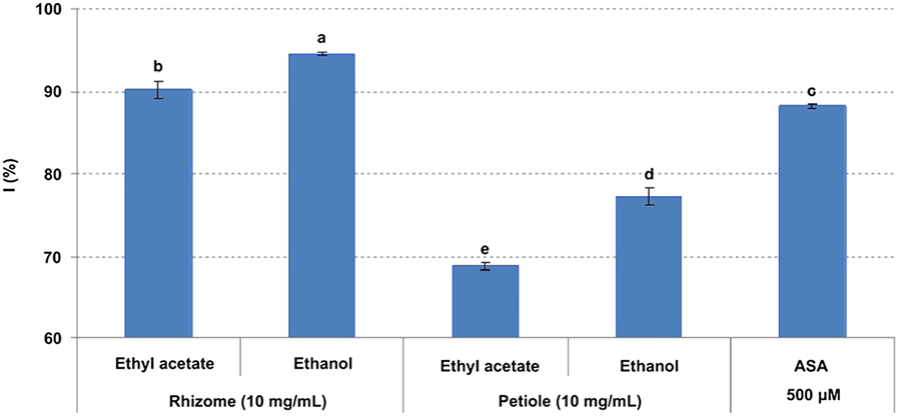
**I%of extracts from *P.hexandrum *measured by the DPPH assay.** ASA was tested as a positive control. The data are the mean of triplicate measurements. Different letters on top of the column were considered significant at *p* < 0.01.

**Figure 2:**
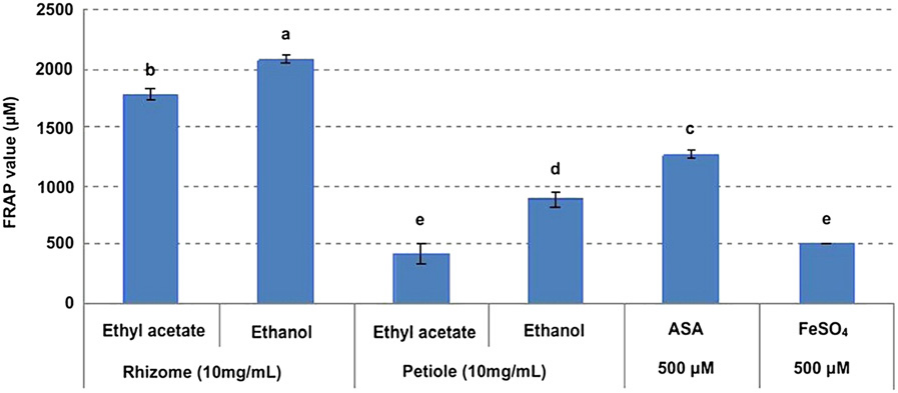
**FRAP value of extracts from *P.hexandrum *measured by the FRAP assay.** ASA was tested as a positive control. The data are the mean of triplicate measurements. Different letters on top of the column were considered significant at p < 0.01.

FRSAs from the rhizome and petiole extracts of *P*. *hexandrum* expressed as I % are showed in Figure 1, and the antioxidant capacity expressed as FRAP value is reported in Figure 2. FRSAs of ethyl acetate and ethanol extracts were higher from the rhizome (90.17 ± 1.11 and 94.52 ± 0.17%, respectively) than from the petiole (68.75 ± 0.52 and 77.23 ± 1.01%, respectively). The FRAP values showed the same trend as FRSAs, and the FRAP values of ethyl acetate and ethanol extracts from the rhizome were 1784.09 ± 52.07 and 2079.55 ± 34.09 μM, and the values of the petiole were 420.45 ± 85.79 and 886.36 ± 68.18 μM. In both DPPH and FRAP assays, 500 μM ASA was tested as a positive control, and the values of I% and FRAP value were 88.25 ± 0.24% and 1267.5 ± 30.24 μM, respectively. The statistical analyses showed that the rhizome extracts had greater antioxidant capacity than the petiole extracts and the ethanol extracts had greater antioxidant capacity than the ethyl acetate extract both in DPPH and FRAP assays (*p* < 0.01).

### Separation and identification of rhizome extracts

**Figure 3:**
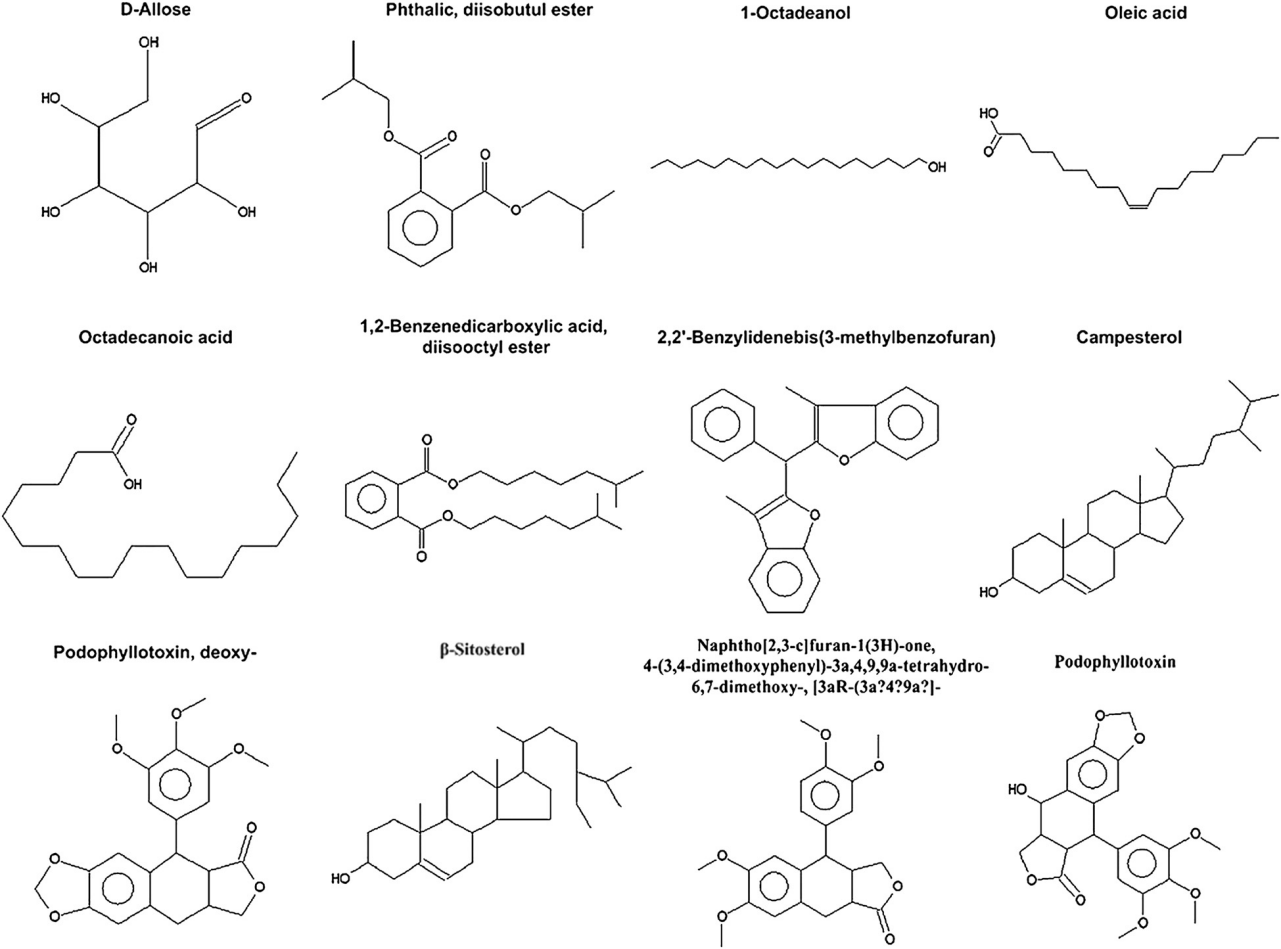
**Compound structures of ethyl acetate extracts from *P.hexandrum *rhizome, determined by GC-MS.**

**Figure 4:**
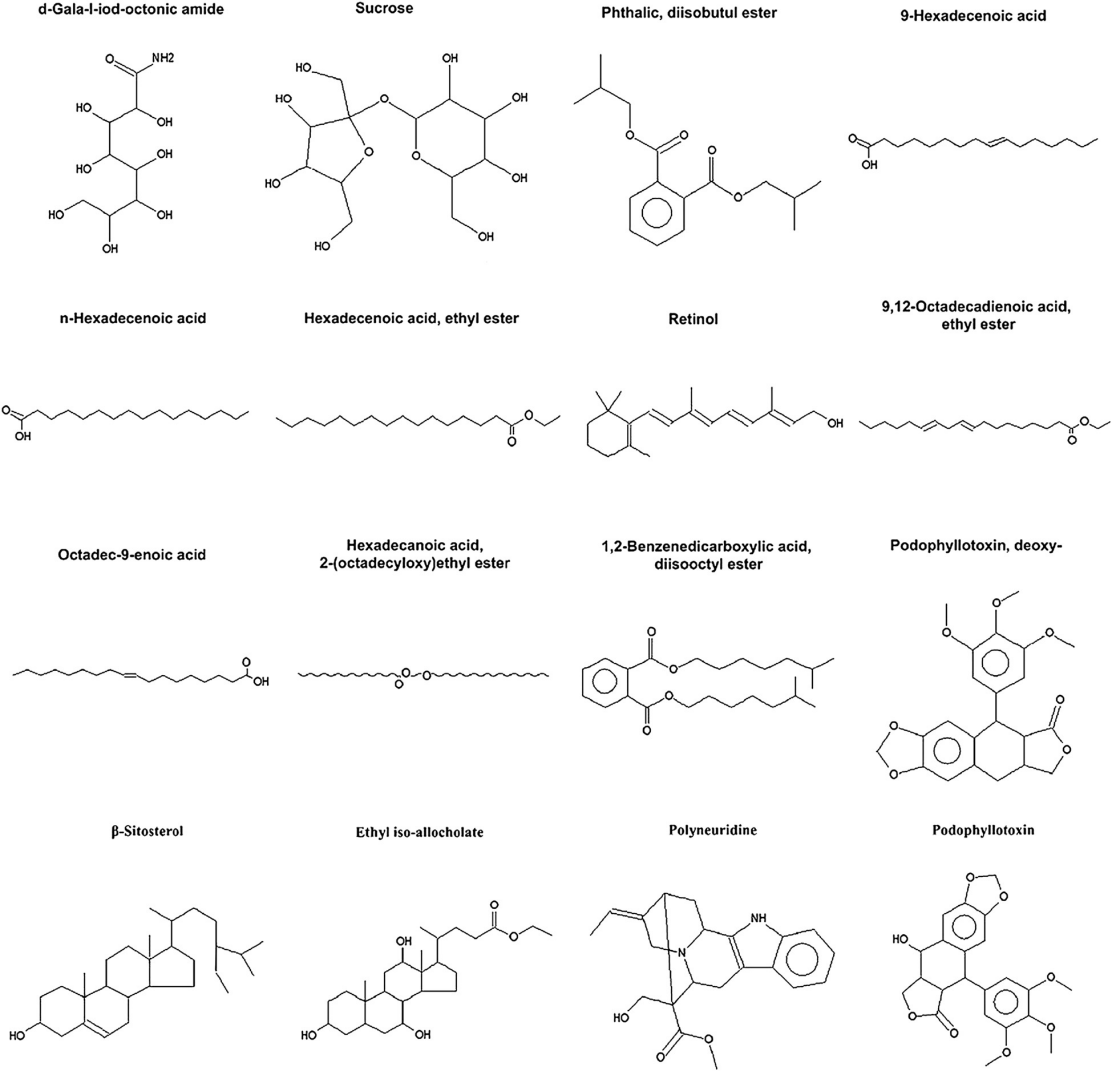
**Compound structures of ethanol extracts from *P.hexandrum *rhizome, determined by GC-MS.**

In order to further study the biochemical compositions in *P*. *hexandrum* rhizome, the extracts of the ethyl acetate and ethanol were separated and identified by GC-MS. In total, About 16 kinds of reactive oxygen were identified. The compound retain time (RT), name, molecular formula, molecular weight, area and percentage of area (POA) were listed in Table 1 (the ethyl acetate extract) and Table 2 (the ethanol extract), and the compound structures were presented in Figure 3 (the ethyl acetate extract) and Figure 4 (the ethyl acetate extract).

**Table 1:** Separation and identification of ethyl acetate extract from ***P *. ***hexandrum *rhizome by GC-MS

**No**.	**RT**	**Name**	**Molecular Formula**	**molecular weight**	**Area**	**POA(%)**
1	16.56	D-Allose	C_6_H_10_O_5_	162	68174247.68	1.85
2	20.24	Phthalic acid, diisobutyl ester (PADE)	C_16_H_22_O_4_	278	101990836.36	2.77
3	22.44	1-Octadecanol	C_18_H_38_O	270	5668336.18	0.15
4	22.96	Oleic Acid	C_18_H_34_O_2_	282	57474673.65	1.56
5	23.17	Octadecanoic acid	C_18_H_36_O_2_	284	15872716.84	0.43
6	26.29	1,2-Benzenedicarboxylic acid, diisooctyl ester (BADE)	C_24_H_38_O_4_	390	82848910.41	2.25
7	30.56	2,2'-Benzylidenebis(3-methylbenzofuran)	C_24_H_20_N_2_O	352	18407733.56	0.50
8	35.04	Campesterol	C_28_H_48_O	400	8076867.21	0.22
9	35.89	Podophyllotoxin, deoxy (PODD)	C_22_H_22_O_7_	398	758866242.2	20.59
10	37.19	β-Sitosterol	C_29_H_50_O	414	59476314.62	1.61
11	38.05	Naphtho[2,3-c]furan-1(3H)-one,4-(3,4-dimethoxyphenyl)-3a,4,9,9a-tetrahydro-6,7-dimethoxy-, [3aR-(3a?4?9a?]-	C_22_H_24_O_6_	384	24682996.63	0.67
12	40.77	Podophyllotoxin (Podofilox) (POD)	C_22_H_22_O_8_	414	2484773776	67.41

**Table 2:** **Separation and identification of ethanol extract from *P*. *hexandrum *rhizome by GC-MS**

**No**.	**RT**	**Name**	**Molecular formula**	**Molecular weight**	**Area**	**POA (%)**
1	14.25	d-Gala-l-ido-octonic amide	C_8_H_17_NO_8_	255	651876.57	0.75
2	16.94	Sucrose	C_12_H_22_O_11_	342	2221794.92	2.55
3	20.75	Phthalic acid, diisobutyl ester (PADE)	C_16_H_22_O_4_	278	1067190.30	1.23
4	21.12	9-Hexadecenoic acid	C_16_H_30_O_2_	254	577281.13	0.66
5	21.81	n-Hexadecanoic acid	C_16_H_32_O_2_	256	4151490.41	4.77
6	22.04	Hexadecanoic acid, ethyl ester	C_18_H_36_O_2_	284	2383067.34	2.74
7	22.81	Retinol	C_20_H_30_O	286	660693.34	0.76
8	23.64	9,12-Octadecadienoic acid, ethyl ester	C_20_H_36_O_2_	308	2368466.98	2.72
9	23.82	Octadec-9-enoic acid	C_18_H_34_O_2_	282	1549676.99	1.78
10	26.52	Hexadecanoic acid, 2-(octadecyloxy) ethyl ester	C_36_H_72_O_3_	552	1112593.91	1.28
11	26.78	1,2-Benzenedicarboxylic acid, diisooctyl ester (BADE)	C_24_H_38_O_4_	390	6141357.57	7.05
12	35.99	Podophyllotoxin, deoxy (PODD)	C_22_H_22_O_7_	398	18255949.66	20.97
13	37.60	β-Sitosterol	C_29_H_50_O	414	1063814.09	1.22
14	39.00	Ethyl iso-allocholate	C_26_H_44_O_5_	436	673221.09	0.77
15	40.01	Polyneuridine	C_21_H_24_N_2_O_3_	352	24387006.54	28.01
16	40.23	Podophyllotoxin (Podofilox) (POD)	C_22_H_22_O_8_	414	19787459.99	22.73

The ethyl acetate and ethanol extracts contained five common components: PADE, BADE, PODD, β-Sitosterol, POD, and the POA of the five components in the ethyl acetate and ethanol extracts were 2.77 and 1.23, 2.25 and 7.05, 20.59 and 20.97, 1.61 and 1.22, 67.41 and 22.73%, respectively. The Polyneuridine also showed the high POA with 28.01% in the ethanol extract.

### Evaluation of antioxidant capacity

**Figure 5:**
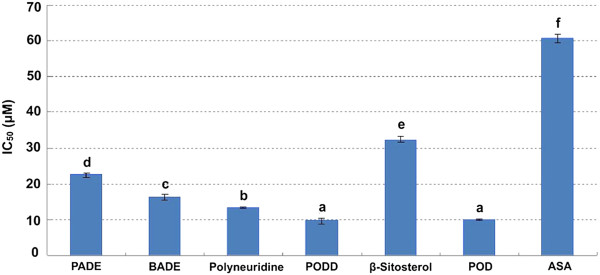
**IC_50_ of the 6 components in the extracts from *P. hexandrum *rhizome.** ASA was tested as a positive control. The data are the mean of triplicate measurements. Different letters on top of the column were considered significant at *p* < 0.01.

**Figure 6:**
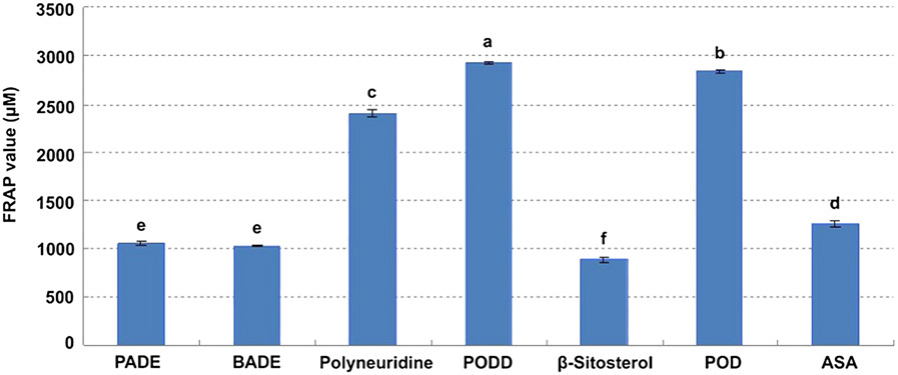
**FRAP value of the 6 components in the extracts from *P. hexandrum *rhizome.** ASA was tested as a positive control. The data are the mean of triplicate measurements. Different letters on top of the column were considered significant at *p* < 0.01.

Figure 5 and 6 show antioxidant capacity of the six components identified from the extracts by using the DPPH and FRAP assays at the same concentration of 500 μM. The IC_50_ and FRAP values of PADE, BADE, Polyneuridine, PODD, β-Sitosterol, and POD were 22.49 ± 0.60 and 1062.5 ± 23.39 μM, 16.32 ± 0.67 and 1025.00 ± 9.24 μM, 13.37 ± 0.35 and 2404.32 ± 36.88 μM, 9.61 ± 0.81 and 2923.98 ± 21.89 μM, 32.43 ± 0.81 and 891.37 ± 22.14 μM, 9.98 ± 0.24 and 2847.27 ± 14.82 μM, respectively. Both of the tested statistics of IC_50_ and FRAP values showed that PODD, POD and Polyneuridine had greater antioxidant capacity than the positive control ASA (60.78 ± 1.22 and 1267.5 ± 30.24 μM) (*p* < 0.01).

## Discussion

*Podophyllum* contained 4–20% of podophyllum resin, which is the source of POD [24,25]. Giri *et al*. [3] reported that Podophyllotoxin is commonly extracted from *P*.*hexandrum* that contains 6–12% of resin, of which the concentration of podophyllotoxin is around 40%. Chawla *et al*. [26] reported that polyphenols and lignans were rich in *P*.*hexandrum* rhizome, which revealed several bioactivities of direct relevance to radioprotection.

In this work, about 16 kinds of reactive oxygen components were identified by GC-MS, some of which have been preliminarily studied for antioxidant capacity or identified from other plants. Zhang *et al*. [27] and Jin *et al*. [28] reported that POD derivatives had strong antioxidative activities. Ng *et al*. [29] revealed that PODD could inhibit lipid peroxidation in brain and kidney homogenates, and the compound had antioxidant effects. Besides antioxidant potential, β-Sitosterol exhibited a protective action against DMH-induced depletion of antioxidants, such as catalase, superoxide dismutase, and glutathione peroxidase [30]. Zhao *et al*. [31] identified PADE from traditional Chinese medicines of pungent flavor and cool nature by GC-MS. Lin *et al*. [32] isolated BADE from the volatile components of *Eclipta prostrate* by GC-MS. The six components PADE, BADE, Polyneuridine, PODD, β-Sitosterol, and POD presented high POA (pereentage of area) in the extracts, and the highest POAs of PODD, POD and Polyneuridine were 20.97, 67.41 and 28.01% in the rhizome, respectively, these results suggest that the components PODD, POD and Polyneuridine are very critical for the antioxidant capacity.

Although the POAs of other components in the ethyl acetate and ethanol extracts are relatively small, antioxidant capacity was also reported in other plant extracts, such as D-allose [33], Dibutyl phthalate [34,35], n-Hexadecanoic acid [33-36], Oleicacid [36,37], Octadecanoic acid [37], Octadec-9-enoic acid [32], Campesterol [38,39], Retinol [40]. So far, both PADE and BADE have not been reported on the antioxidant capacity. Other active components might be present in the extracts and should be under further investigation.

## Conclusions

The rhizome extracts had greater antioxidant capacity than the petiole extracts in DPPH and FRAP assays. About 16 kinds of reactive oxygen components were identified by GC-MS in the extracts of *P*.*hexandrum*, this was the first to report PADE and BADE from a plant product and further study the antioxidant capacity of the identified components PADE, BADE, Polyneuridine, PODD, β-Sitosterol, and POD. The results of tested data and statistical analysis proved that the extracts from *P*. *hexandrum* had strong antioxidant capacity, and the PODD, POD and Polyneuridine played an important role. Based on the above data it can be recommended as an alternative plant material of antioxidant and radical scavenging activity.

## Competing interests

The authors declare that they have no competing interests.

## Authors’ contributions

MFL designated the study, performed the statistical analysis and drafted the manuscript. LLZ and TTL participated in the extraction and the antioxidant assays. DLY did the isolation and structure elucidation part and helped in manuscript editing. WL has made substantial contribution to conception and design, and revising the manuscript for intellectual content. All authors read and approved the final manuscript.

## Pre-publication history

The pre-publication history for this paper can be accessed here:

http://www.biomedcentral.com/1472-6882/12/263/prepub
